# Effects of Non-Nutritive Artificial Sweeteners on Gut Microbiota and Host Metabolism and Health-Related Outcomes: A Review

**DOI:** 10.3390/nu18142402

**Published:** 2026-07-22

**Authors:** Yefu Xin, Tianzhizi Fu, Ralf Weiskirchen, Han Chen, Mengyu Cheng, Rui Zhu, Hualin Wang

**Affiliations:** 1 School of Life Science and Technology, Wuhan Polytechnic University, Wuhan 430023, China; garyxin200319@outlook.com (Y.X.); ftzz_freya@163.com (T.F.); 15027359181@163.com (M.C.); 2 School of Life Science, The Chinese University of Hong Kong, Hong Kong SAR 999077, China; 3 Institute of Molecular Pathobiochemistry, Experimental Gene Therapy and Clinical Chemistry (IFMPEGKC), RWTH University Hospital Aachen, D-52074 Aachen, Germany; 4 School of Life Science, Fujian Agriculture and Forestry University, Fuzhou 350002, China; 17786753445@163.com; 5 Department of Integrated Chinese and Western Medicine, Union Hospital, Tongji Medical College, Huazhong University of Science and Technology, Wuhan 430022, China

**Keywords:** non-nutritive artificial sweeteners (NAS), gut microbiota, host metabolism, health-related outcomes, NAS-MAP knowledge graph

## Abstract

As obesity, diabetes and other diet-related metabolic disorders continue to rise, non-nutritive artificial sweeteners (NASs) are widely used as sugar substitutes in free-sugar reduction and weight-management strategies, but their effects on the gut microbiota, host metabolism and related health outcomes remain debated. To clarify this evidence landscape, this review synthesizes current knowledge on the effects of major NAS on the gut microbiota and host metabolic and health-related outcome. A bibliometric analysis was conducted to characterize the development, knowledge structure and thematic evolution of this research area. For the eight NAS exposure categories included in this review (saccharin, cyclamate, aspartame, acesulfame potassium, sucralose, neotame, neohesperidin dihydrochalcone and mixed NAS), we performed a compound-specific analysis integrating physicochemical characteristics, regulatory status, and research findings from in vitro, ex vivo, animal and human studies on gut microbiota alterations, host metabolic and health-related outcomes. These findings were further compared through four interpretive dimensions: exposure conditions, research model background, outcome assessment and interpretation of discordant results. Current research evidence does not support a class-wide conclusion that NAS are uniformly harmful or safe. To make research findings searchable, we developed the NAS-MAP knowledge graph, an interactive non-nutritive artificial sweetener–microbiota-associated phenotype knowledge graph. Overall, this review consolidates current knowledge, clarifies the sources of inconsistent findings, and provides a framework for more mechanistically informative studies, standardized evidence reporting, and more precise sweetener-, exposure- and population-specific dietary and public health guidance.

## 1. Introduction

The global burden of obesity and diabetes continues to rise, intensifying efforts to reduce free sugar intake across food systems and clinical care [1,2]. Non-nutritive artificial sweeteners (NASs) occupy a prominent but contested position within these strategies because they preserve sweetness while contributing little or no energy. The conditional recommendation issued by the WHO against using non-sugar sweeteners for long-term weight control underscores an important distinction: lower caloric content does not itself establish sustained health benefit. NASs cannot be treated as a single exposure. Individual compounds differ in chemical structure, sweetness potency, absorption, intestinal availability, technological use, and formulation context [3,4]. Therefore, users encounter a defined compound or mixture within a dietary matrix, not an abstract sensation of sweetness. This distinction is especially important when gut microbiota may transform dietary exposures into signals that conventional nutrient and toxicological categories do not capture. The central question is consequently not whether NASs are uniformly beneficial or harmful, but which compounds produce biologically meaningful effects under specific exposure and host conditions.

Gut microbiota research has expanded this question from taxonomic description to functional and mechanistic interpretation. Human multi-omics evidence indicates that gut microbial carbohydrate metabolism, metabolite production and community interaction patterns can contribute to host metabolic regulation, including insulin resistance and glycemic variability [5]. Recent syntheses on NASs have broadened the field beyond sweet taste perception and energy balance, highlighting intestinal taste receptor signaling, epithelial transport, gut microbial growth, quorum sensing, metabolite exchange and compound-specific bacterial physiology as plausible response layers [4,6,7,8]. These pathways are not mutually exclusive: a sweetener may be poorly absorbed yet still encounter intestinal microbes, may affect bacterial behavior without producing broad taxonomic restructuring, or may alter host physiology through epithelial or endocrine mechanisms that only partially depend on gut microbial mediation. NAS research has also generated landmark and translationally relevant findings. The original gut microbiota transfer study linking NASs to glucose intolerance remains foundational because it established a testable gut microbial mechanism [9]. Later human intervention work demonstrated personalized microbiome and glycemic responses across several sweeteners [10]. Together, these findings justify evaluating NAS through linked but distinct evidence layers: compound identity, gut microbial composition, gut microbial function, host phenotype, and causal support.

However, the evidence remains insufficiently coherent for a class-wide conclusion. Controlled human studies illustrate the problem. In healthy adults, sucralose exposure was associated with altered glucose homeostasis and gut microbiota-related features [11]. By contrast, two 12-week randomized trials examined Asian Indian adults with overweight, obesity, or type 2 diabetes. Replacing added sugars with sucralose produced no major restructuring of gut microbiome composition [12]. The multicenter SWEET trial added a different perspective. Prolonged replacement with sweeteners and sweetness enhancers supported weight loss maintenance and altered overall gut microbiota composition without major cardiometabolic deterioration [13]. Comparative long-term experiments likewise reported predominantly neutral or context-dependent metabolic effects across several sweeteners [14]. These studies address different compounds, formulations, comparators, populations, durations, and endpoint layers. These differences also change the inferential strength of each observation. A compositional shift may be biologically active without mediating the measured host outcome, while an unchanged diversity metric may coexist with functional or metabolic change. Their divergence should not be reduced to positive versus negative findings. It instead exposes the need to distinguish purified compounds from commercial mixtures, composition from function, association from mediation, and detectable biological activity from clinically meaningful harm or benefit.

A renewed synthesis is now warranted because the evidentiary unit of the field has changed. Recent European consortia now provide complementary acute, short-term, and year-long randomized evidence across weight management, metabolic biomarkers, sweet taste exposure, and gut microbiota [15]. A recent mechanistic study moved beyond community-level associations by showing that saccharin can directly disrupt bacterial envelope stability, DNA replication dynamics, motility, and biofilm formation [16]. A targeted multicohort study quantified specific sweeteners across stool, urine, and serum. Measured exposure was associated with Crohn’s disease activity across cohorts [17]. In parallel, recent food science research treats sugar replacement as a matrix-level intervention shaped by ingredient interactions, processing, sensory performance, and safety evaluation [18]. These frameworks indicate that sweetener identity alone is insufficient for interpreting newer formulations [19]. Together, these developments shift the central question from whether NAS alter the gut microbiota to when a defined exposure produces a reproducible microbial and host response. Taken separately, domain-specific syntheses may not resolve how these evidence layers relate. This review therefore integrates compound-specific evidence across experimental scales and evaluates heterogeneity through a connected chain from formulation and exposure to microbial response and host metabolic outcome. Such integration is needed now to distinguish reproducible biological signals from effects generated by incomparable exposures, endpoints, or study contexts.

This review synthesizes evidence on the interactions between major NASs, the gut microbiota, and host metabolic and health-related outcomes, with an emphasis on compound-specific effects and the experimental conditions under which they arise. Bibliometric mapping is used to reconstruct the development, intellectual structure, and thematic evolution of the field, thereby providing a broader context in which shared patterns, compound-level differences, and persistent knowledge gaps can be identified. Building on this field-level perspective, the review integrates the physicochemical properties, metabolic fate, exposure characteristics, and regulatory context of individual NASs with evidence from in vitro, ex vivo, animal, and human studies. Particular attention is given to how sweetener identity, dose, exposure matrix, duration, host background, study design, and the choice of microbial or physiological endpoints shape apparently divergent findings. By distinguishing changes in gut microbial composition from functional consequences, host-level outcomes, and direct causal support, the review provides a structured framework for evaluating the biological and translational significance of current evidence. This integrated analysis ultimately informs priorities for improved exposure assessment, mechanistic validation, clinically relevant population studies, functional and multi-omics microbiome profiling, and standardized reporting, with the aim of advancing the field towards evidence capable of supporting robust dietary and public health guidance.

## 2. Methodology

### 2.1. Literature Search and Bibliometric Overview

**Figure 1:**
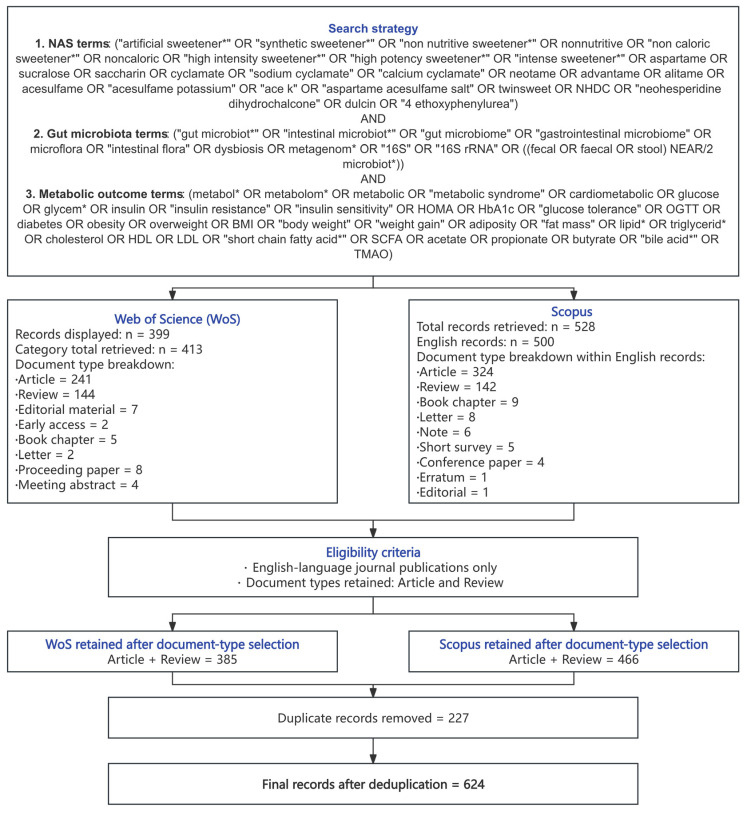
Literature identification and deduplication workflow used for the bibliometric overview of the field.

The Web of Science Core Collection and Scopus were searched in March 2026 without a lower publication date limit. Searches were restricted to English-language journal articles and reviews and combined terms covering non-nutritive artificial sweeteners, gut microbiota, and metabolic or health-related outcomes. Database-specific field tags and proximity operators were adapted according to the syntax supported by each database. The record identification and deduplication workflow used for the bibliometric overview is shown in Figure 1. After applying the language and document-type restrictions, 385 records from Web of Science and 466 records from Scopus were retained. The records were merged, and removal of 227 duplicates produced a bibliometric dataset of 624 unique publications. VOSviewer version 1.6.20 was used to construct keyword co-occurrence and reference co-citation networks. R version 4.5.1 was used for descriptive bibliometric statistics, burst detection, trend topic profiling, publication growth modeling, and mapping of country-level research output and international collaboration. The bibliometric overview was used to describe the development, knowledge structure, thematic evolution, and international research distribution of the field. It was not treated as an independent source of biological or clinical evidence.

### 2.2. Evidence Selection and Narrative Synthesis

Following the completion of the bibliometric analysis, topic-relevant publications identified through PubMed, the Web of Science Core Collection, and Scopus were screened for the narrative synthesis. PubMed records were cross-checked against those retrieved from the other databases, and no additional eligible publications were identified in the updated search. Based on the literature search and source selection described above, candidate publications were further assessed against the thematic scope of this review, which focused on reported links among NAS exposure, gut microbiota-related changes, and host metabolic or health-related outcomes. Studies were excluded from the main synthesis when they: (i) examined sugar alcohols or natural sweeteners outside the defined NAS scope; (ii) focused exclusively on sensory properties, food formulation, or analytical chemistry; (iii) evaluated general bacterial toxicity without gut-relevant microbiota endpoints; or (iv) reported host outcomes without microbiota-related assessment, except when cited as background to clarify metabolic or safety-related boundaries. General bacterial toxicity assays using non-gut or engineered reporter strains were excluded when they did not assess gut microbial composition, microbial community function, microbiota-derived metabolites, or microbiota-linked host outcomes. Studies reporting host outcomes without microbiota-related assessment were not treated as evidence for microbiota-mediated effects. Relevant in vitro, ex vivo, animal, and human studies were synthesized by sweetener identity and evidence layer. Findings were interpreted across exposure conditions, research model background, outcome assessment, and the consistency or inconsistency of microbial and host phenotypic responses where both types of outcomes were available. Because this article was designed as a narrative review, formal risk-of-bias assessment, evidence grading, and quantitative meta-analysis were not performed.

### 2.3. NAS-MAP as a Literature-Derived Evidence Organization Framework

To support evidence traceability and structured interpretation of heterogeneous findings, NAS-MAP was proposed as a literature-derived evidence organization and navigation framework. This framework organizes relationships among NAS exposure, experimental context, gut microbiota-related findings, host phenotypes, and DOI-linked source literature. A preliminary implementation was developed using only evidence from publications included and discussed in this review. All records were extracted from the reviewed publications by designated team members and subsequently cross-checked by other team members before inclusion in the web platform. The platform was also developed with a back-end management system to facilitate future updates, including the incorporation of newly published studies and additional curated evidence records. NAS-MAP does not contain unpublished experimental, clinical, animal study, or participant-level data. It also does not generate new analytical results or conclusions beyond those reported in the reviewed literature. Accordingly, NAS-MAP should be interpreted as a preliminary evidence organization framework that supports transparency, source-level traceability, and future machine-readable reporting, rather than as an independent dataset, quantitative evidence-grading system, or comprehensive database.

## 3. Bibliometric Context of Research on NAS, Gut Microbiota, and Host Metabolic Health

**Figure 2:**
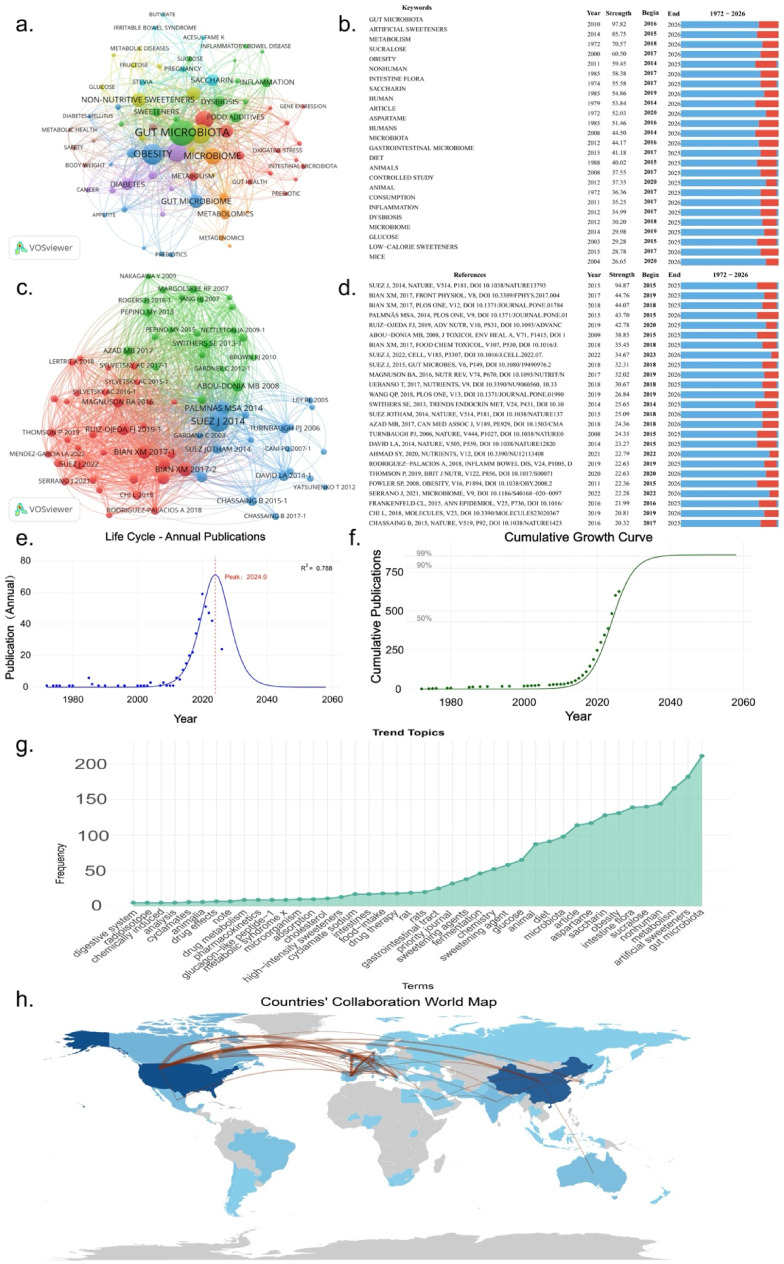
Bibliometric landscape of research on NAS, gut microbiota, and host metabolic health. (**a**) Keyword co-occurrence network; (**b**) keyword burst analysis across 1972–2026; (**c**) reference co-citation network; (**d**) reference burst analysis across 1972–2026; (**e**) life cycle model of annual publication output; (**f**) cumulative logistic growth curve of publication output; (**g**) trend topic frequency distribution; (**h**) world map of country-level research output and international collaboration world map.

The keyword co-occurrence network indicates that gut microbiota-centered terminology forms the dominant conceptual core of the field, with “gut microbiota” serving as the principal hub (Figure 2a). Closely related labels such as “microbiome,” “gut microbiome,” and “microbiota” should be interpreted as overlapping indexing terms that collectively reflect sustained attention to the intestinal microbial ecosystem. Around this microbial core, the network connects artificial sweetener exposure terms, especially “artificial sweeteners” and “saccharin,” with metabolic and pathophysiological concepts including “obesity,” “diabetes,” “metabolism,” “inflammation,” and “dysbiosis.” Omics-related terms such as “metabolomics” and “metagenomics” further suggest that the field has increasingly moved from descriptive exposure assessment toward mechanism-oriented profiling of microbial composition and function. The keyword burst analysis supports this interpretation, showing that “gut microbiota,” “artificial sweeteners,” “metabolism,” “sucralose,” “saccharin,” “aspartame,” “low-calorie sweeteners,” “consumption,” “mice,” and human or nonhuman study contexts remained active research-front terms, with several bursts extending to 2026 (Figure 2b). Together, these patterns indicate a shift from broad safety concerns toward a more mechanistic literature focused on how specific sweeteners may interact with gut microbial ecology and host metabolic regulation. At the reference level, the knowledge base is not defined by isolated landmark papers, but by several converging research traditions. These include gut microbiota and personalized-response studies linking non-nutritive sweeteners to glucose tolerance [9,10], compound- and model-specific work on aspartame, sucralose, acesulfame potassium, and sucralose-containing formulations [20,21,22], clinical or experimental syntheses evaluating sweetener–gut microbiota evidence and biological fate [23,24], human interventions reporting either personalized responses or minimal effects under defined short-term exposure conditions [25,26], and broader microbiome studies connecting diet, obesity, additives, inflammation, and metabolic phenotypes [27]. The reference burst pattern therefore suggests a field anchored in both sweetener-specific mechanistic evidence and the wider gut microbiome–metabolism literature, consistent with the heterogeneity emphasized throughout this review (Figure 2c,d).

The publication output model suggests rapid expansion followed by possible model-predicted maturation, but the fitted curves should be interpreted descriptively rather than as a firm life cycle forecast. Annual publications remained sparse for several decades, increased sharply after the 2010s, and reached a fitted peak in 2024 with moderate fit (R^2^ = 0.788) (Figure 2e). Consistently, the cumulative logistic curve shows an S-shaped trajectory and visually approaches a plateau after approximately 2040, suggesting that the field may shift from accelerated growth toward saturation in the coming decades if current publication patterns persist (Figure 2f).

However, recent expanding multi-omics methods, precision nutrition designs and renewed regulatory interest could all alter the trajectory [28]. In addition, trend topic frequencies reinforce the same structure, with high-frequency terms clustering around gut microbiota, artificial sweeteners, metabolism, sucralose, obesity, saccharin, aspartame, diet, animal models, and glucose-related outcomes (Figure 2g). Country-level mapping shows that output is led by the United States and China, with additional contributions from Canada, Spain, Mexico, the United Kingdom, Germany, India, Italy, Australia, Israel, France, Brazil, and South Korea. International collaboration is concentrated across North America and Europe, with expanding links involving China, India, Australia, and other Asia-Pacific contributors (Figure 2h). Overall, the bibliometric landscape suggests that NAS research is best interpreted by compound identity, exposure form, host model, gut microbiota endpoint, and metabolic outcome rather than as evidence for a uniform biological class effect.

## 4. Overview of Major NASs

NASs are used to replace added sugars because they provide intense sweetness at low use levels and usually contribute little or no energy to the diet [4]. In the broader food additive landscape, commonly discussed NAS include saccharin, cyclamate, aspartame, acesulfame potassium (Ace-K), sucralose, neotame, advantame, and neohesperidin dihydrochalcone (NHDC), which has also attracted increasing attention as a high-intensity sweetener derived through the chemical transformation of a natural precursor.

This review focuses on seven sweeteners: saccharin, cyclamate, aspartame, Ace-K, sucralose, neotame, and NHDC, together with studies of mixed NAS exposures. Advantame is a well-recognized high-intensity NAS. As shown in Figure 1, advantame was included in the search strategy and was not excluded during literature identification. However, screening and data extraction of records published up to March 2026 identified no eligible primary studies directly examining advantame in relation to gut microbiota and associated host metabolic or health-related outcomes. Accordingly, although advantame was covered by the literature search, it was not included in the compound-specific synthesis of this review. The current literature is concentrated mainly on sucralose, aspartame, saccharin and Ace-K, whereas evidence for cyclamate, neotame, and NHDC remains comparatively limited. Based on data compiled from PubChem (https://pubchem.ncbi.nlm.nih.gov/, accessed on 22 June 2026), the U.S. Food and Drug Administration (FDA) (https://www.fda.gov/food/food-additives-petitions/aspartame-and-other-sweeteners-food, accessed on 22 June 2026), the European Food Safety Authority (EFSA) (https://www.efsa.europa.eu/en/topics/topic/sweeteners, accessed on 22 June 2026), and the JECFA database (https://apps.who.int/food-additives-contaminants-jecfa-database/, accessed on 22 June 2026), we summarized the chemical formula, molecular weight, water solubility, thermal properties, relative sweetness versus sucrose, regulatory status, and acceptable daily intake of the major NAS included in this review (Table 1). These NAS differ in molecular structure, molecular weight, water solubility, thermal stability and relative sweetness. These physicochemical differences are closely related to their technological properties and practical use in food systems. Sweeteners with good water solubility and relatively high processing stability, such as sucralose and Ace-K, are generally more suitable for beverages and other liquid formulations, whereas the lower thermal stability of aspartame may limit its application in products exposed to intensive heat treatment. Cyclamate has comparatively lower sweetening potency, while neotame and NHDC exhibit much higher sweetness intensity, which in turn affects use levels, blending strategies and formulation design in food applications. Such differences indicate that these sweeteners are not fully interchangeable in food manufacture, even though they are often discussed within the same broad category.

These differences are also reflected in their regulatory evaluation and permitted use. The FDA, EFSA, and JECFA have established compound-specific safety assessments and, for most major sweeteners, corresponding acceptable daily intakes (ADI) to support safe consumption under approved conditions of use. Regulatory pathways, however, are not fully aligned across jurisdictions. Cyclamate remains prohibited in the United States, whereas NHDC entered the U.S. market through the GRAS (Generally Recognized as Safe) route and is also recognized within the European regulatory framework. Therefore, NAS should not be interpreted as a fully uniform category when their biological effects are discussed. A more meaningful understanding requires compound-specific consideration, particularly with respect to chemical identity, exposure level, regulatory status, and conditions of use, as these factors may all influence their interactions with the gut microbiota and the resulting host-related outcomes.

## 5. Effects of Individual NASs on Gut Microbiota and Host Metabolic and Related Health Outcomes

### 5.1. Experimental Models and Research Approaches

**Figure 3:**
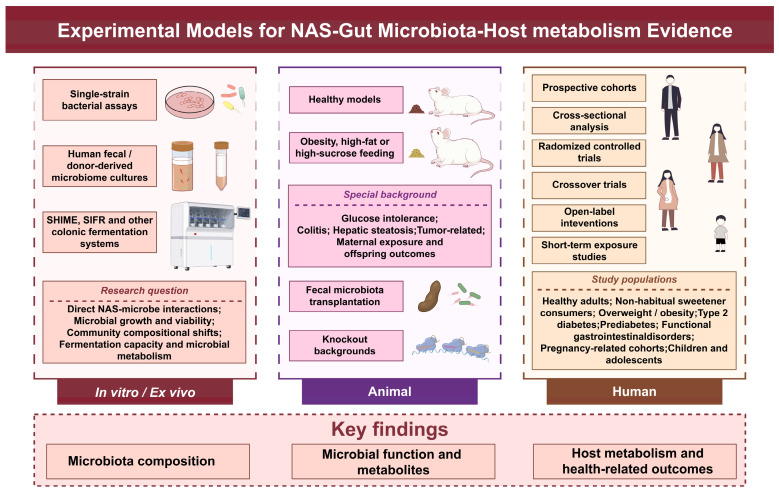
Experimental models for studies of NASs, gut microbiota and host-related outcomes.

The current evidence base is derived mainly from studies using a single model system, most commonly in vitro, animal or human designs, whereas studies integrating more than one experimental layer remain comparatively limited. In vitro and ex vivo approaches, including single-strain assays, epithelial co-cultures, fecal fermentation systems, SHIME- or SIFR-type platforms, and simplified communities are useful for identifying direct NAS–microbe interactions, community shifts, fermentation outputs, quorum-sensing effects, and plasmid transfer phenotypes. They generate mechanistic hypotheses but cannot establish host metabolic consequences. Animal studies are dominated by mice and rats, with additional use of guinea pigs, zebrafish, and *Drosophila*, and cover both healthy and disease-related settings, including obesity, high-fat or high-sucrose feeding, glucose intolerance, colitis, hepatic steatosis, reproductive and developmental outcomes, and tumor- or immunotherapy-associated contexts. In addition, human evidence adds population relevance through prospective cohorts, cross-sectional analyses, randomized controlled trials, crossover trials, open-label interventions, and short exposure studies in healthy adults, non-habitual sweetener users, individuals with overweight, obesity, prediabetes or type 2 diabetes, pregnancy-related cohorts, children and adolescents [9,10,26,29,30] (Figure 3). For questions concerning effects in humans, controlled intervention studies were assigned the greatest translational weight, followed by prospective observational evidence. For questions concerning microbiota-mediated causality, studies incorporating experimental perturbation, fecal microbiota transplantation, gnotobiotic models, rescue experiments, or targeted metabolite validation were considered more informative than those reporting paired associations alone. Ex vivo community models and single-strain assays were used to establish biological plausibility but were not considered sufficient to infer host-level or clinical effects. Accordingly, translational relevance and mechanistic causal support were evaluated as complementary dimensions rather than integrated into a single universal evidence hierarchy.

### 5.2. NAS-Specific Evidence Across Gut Microbiota and Host-Related Health Outcomes

**Figure 4:**
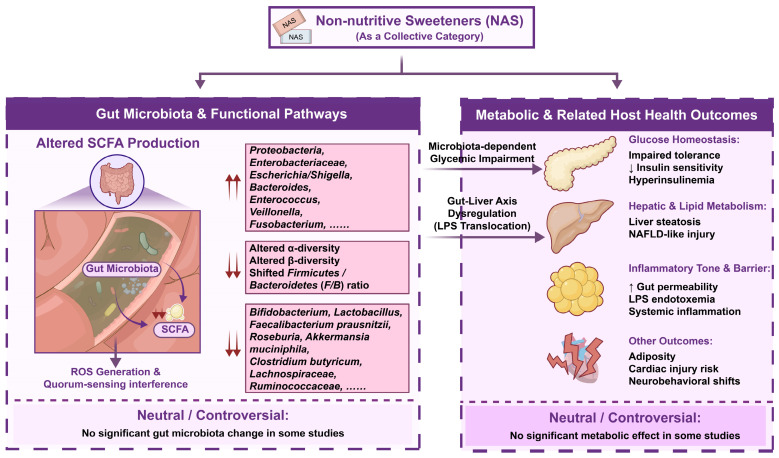
Compound-specific and context-dependent response framework for NAS effects on gut microbiota and host-related outcomes. The figure summarizes how individual NASs and mixed formulations may be linked to gut microbiota composition, gut microbial function or metabolites, and host outcomes such as glycemic regulation, intestinal barrier function, inflammation, hepatic lipid metabolism, adiposity, or neurobehavioral endpoints. Note: arrows and outcome labels should be interpreted as evidence-mapping relationships rather than uniform category-level effects, because reported directions vary by compound, dose, formulation, exposure duration, host background, model system, and endpoint layer.

Evidence on NAS does not support a single class-level conclusion. Across direct microbial assays, ex vivo community systems, animal models and human studies, reported outcomes include shifts in community composition, depletion or enrichment of short-chain fatty acid (SCFA)-associated taxa, changes in microbial metabolites and functional pathways, and host-related phenotypes involving glucose homeostasis, adiposity, intestinal barrier integrity, inflammatory tone, liver injury, developmental programming, neurobehavioral outcomes, and gastrointestinal symptoms. Several studies, however, report little or no measurable effect under the tested conditions. The evidence is therefore best interpreted by compound identity, exposure context, study layer, and endpoint layer, rather than by treating NAS as a biologically uniform class (Figure 4).

#### 5.2.1. Sucralose

Sucralose is one of the most frequently studied NASs, but its gut microbiota profile remains context-dependent rather than uniformly disruptive. In human fecal and ex vivo systems, sucralose reduced *Roseburia* and *Faecalibacterium prausnitzii* and increased taxa such as *Enterococcus*, *Veillonella*, *Mucispirillum schaedleri*, *Escherichia-Shigella* and *Bilophila*, with parallel changes in valerate production and other fermentation-related outputs [31,32]. Animal and human studies have reported lower *Lactobacillus*, *Bifidobacterium*, *Faecalibacterium prausnitzii*, *Lachnospiraceae*-related genera, or *Akkermansia muciniphila*, together with enrichment of *Bacteroides*, *Clostridium*, *Proteobacteria*-related taxa, *Prevotella copri* or other inflammation-associated lineages in selected settings [21,33,34,35,36,37]. These compositional findings were frequently accompanied by lower butyrate availability or reduced butyrate-producing capacity [11,38,39,40], altered bile acid signaling and gut microbiota-derived metabolites [33], and direct gut microbial functional effects including quorum-sensing interference, bacteriostatic activity, and enhanced plasmid-mediated conjugative transfer [41]. Product-based exposure showed a similarly mixed pattern. In young mice, Splenda^®^ (main NAS: sucralose) was associated with marked changes in cultured small-intestinal microbiota together with immune-related alterations after chronic exposure [42], whereas Sweeny^®^ Plus (main NAS: sucralose), administered at an ADI-equivalent dose, did not significantly alter α-diversity, β-diversity, phylum-level composition or the *Firmicutes*/*Bacteroidetes* ratio (*F*/*B* ratio), although selected genera such as *Romboutsia* and *Lactobacillus* were affected under different dietary backgrounds [43].

These microbial changes were reported together with a wide range of host phenotypes. Several studies linked sucralose exposure with impaired glycemic control, higher insulin or C-peptide responses, reduced insulin sensitivity, altered incretin-related signaling, or gut microbiota-dependent glucose intolerance [10,11,44,45,46]. In other studies, the dominant phenotype involved the gut–liver axis, including higher circulating lipopolysaccharide (LPS), impaired barrier-related indices, altered bile acid metabolism, suppression of gut microbiota-derived aryl hydrocarbon receptor ligands, hepatic inflammation, steatosis, or nonalcoholic fatty liver disease (NAFLD)-like injury [33,35,36,39,47]. Additional work connected sucralose with worsened dextran sulfate sodium (DSS)-induced colitis [21,37,40], offspring adiposity or later steatosis programming after maternal exposure [30], altered ovarian function with antibiotic-sensitive endotoxemia [45], reduced response to cancer immunotherapy [48], altered growth performance and cecal microbiology in rabbits [49], and neurobehavioral change accompanied by microbial disturbance in zebrafish [50]. However, these host outcomes were not uniformly adverse in all settings. Lower body weight gain, increased energy expenditure or improved insulin sensitivity were also reported under some dietary conditions [14].

In addition, several studies reported that sucralose did not produce clear changes in gut microbiota or in host metabolic and health-related outcomes under the tested conditions. In a 48 h ex vivo human gut microbiota platform, sucralose remained largely intact and did not measurably change diversity, cell density, community composition or metabolite production [51]. A short human intervention detected no significant change in fecal microbiota composition or host glycemic indices [52], and another crossover trial found no measurable effect of 14-day pure sucralose consumption on fecal diversity, dominant taxa, fecal SCFAs, glucose metabolism, or insulin sensitivity [25].

#### 5.2.2. Aspartame

Aspartame has been linked more often with altered microbial function, metabolic regulation, and developmental programming. In fermentation or bacterial systems, aspartame increased acetate and propionate production, lowered branched-chain fatty acids, and promoted *Bifidobacterium* growth when delivered as an aspartame-based sweetener in a fiber fermentation model [32]. Aspartame has also been implicated in quorum-sensing inhibition, adhesion-related changes, and enhanced plasmid transfer in mechanistic microbial studies [53,54,55]. In vivo, different settings have reported higher *Enterobacteriaceae*, *Clostridium leptum*, *Roseburia* or *Escherichia-Shigella*, lower *Bifidobacterium* in obesity-related models, and offspring microbial configurations enriched for propionate-related and butyrate-related pathways after maternal exposure [20,29,56,57,58]. Human data have described duodenal or stool microbial changes without overt diversity differences, including lower *Lactobacillus*, *Fusobacterium*, *Escherichia*, *Klebsiella* and *Salmonella* and higher selected environmental or minor taxa [59]. However, this microbial profile remained inconsistent across studies. A broad ex vivo metaproteomic screen indicated only limited global perturbation for aspartame within the target sweetener group [60], a comparative long-term animal study did not identify a stable dominant aspartame-associated gut microbiota pattern [14], and short-term pure aspartame exposure in healthy adults left fecal diversity, dominant genera, SCFAs, and broad gut microbiota structure unchanged [25].

Host-level findings for aspartame are more coherent in susceptible or developmental settings than in short-term healthy adult interventions. In obesity diet or high-fat diet models, aspartame was associated with higher fasting glucose, impaired insulin-stimulated glucose disposal, hyperinsulinemia, worsened glucose tolerance, and adipose inflammation [20,57]. In a DSS-induced colitis mouse model, aspartame altered fecal microbial diversity and community composition under both normal and inflammatory conditions. In the colitis setting, it was accompanied by more severe intestinal injury, higher inflammatory cytokine levels and greater epithelial barrier damage, whereas no obvious gut pathology was observed in healthy mice receiving aspartame alone [61]. However, in a human observational study comparing self-reported aspartame-only consumers with controls, aspartame was associated with reduced serum IL-10 and IL-6 [59]. Developmental and reproductive studies further associated maternal or early-life exposure with altered offspring adiposity, glucose tolerance, and insulin sensitivity [30,56,58]. In addition, one neonatal multi-omics study reported that maternal aspartame exposure was associated with altered gut microbiota-related metabolic signatures in the offspring, together with pulmonary metabolic dysregulation, increased oxidative stress, and inflammasome activation [62]. A further study combining animal and human evidence reported delayed female pubertal onset in rats, together with lower fecal SCFAs and enrichment of *Escherichia*–*Shigella* in the animal model [29]. Nevertheless, human interventions have not consistently reproduced clear metabolic impairment. In the personalized intervention study, aspartame altered gut microbiota and metabolome features but did not produce the group-level glucose intolerance observed with saccharin and sucralose [10]. Another crossover study reported no measurable effect on glucose metabolism or insulin sensitivity [25].

#### 5.2.3. Saccharin

Saccharin has been investigated across mechanistic, animal and human settings, but the reported gut microbiota-related effects vary substantially across models and outcome measures. Direct gut microbiota studies showed that saccharin reduced *Clostridium butyricum*, increased *Escherichia coli*, and disturbed SCFAs and pathway-level outputs [63]. Mechanistic systems also reported quorum-related interference, bacteriostatic behavior, and enhanced plasmid transfer [54,55]. In vivo, saccharin altered community structure or composition in several models, including changes in *Lactobacillus*, *Muribaculaceae*, *Oscillospira*, *Roseburia*, *Turicibacter* and *Akkermansia muciniphila* [34,36,64,65]. Beyond 16S rRNA-based compositional profiling, metabolomic analysis can also capture gut microbiota-related metabolic changes, including bile acid-related alterations [66]. These gut microbiota-related and metabolic alterations were accompanied by a range of host metabolic and health-related outcomes. Reported host outcomes included impaired glycemic responses or glucose intolerance [9,10], higher serum glucose and activation of hypothalamic sweet-signaling pathways [64], increased body weight and triglyceride levels in some product-based exposure settings [34], liver inflammation and related liver injury outcomes [65], and gut–liver signaling abnormalities consistent with NAFLD-like injury [36]. The strongest gut microbiota-mediated support comes from studies that linked saccharin-related microbial changes to glucose intolerance through antibiotics, fecal transfer, or personalized microbiome experiments.

At the same time, saccharin did not consistently reproduce these effects across host backgrounds and intervention designs. High-dose saccharin supplementation in healthy humans and mice produced no significant change in α-diversity, β-diversity, fecal metabolome, SCFAs, or major glycemic endpoints [26]. Early-life NAS exposure also failed to produce robust gut microbiota differences in the saccharin-containing arm, even though disturbances in postprandial glucose handling, sugar motivation, or memory-related outcomes were reported [67].

#### 5.2.4. Ace-K

Ace-K has been associated with gut microbial changes that are often reported together with intestinal inflammation, impaired barrier integrity, and liver-related metabolic disturbance, although the overall pattern is not fully uniform across studies. In healthy mice, Ace-K lowered gut microbial α-diversity and produced clear separation in gut microbial β-diversity. These changes were accompanied by enrichment of *Erysipelotrichaceae* and reductions in *Clostridiaceae*, *Lachnospiraceae* and *Ruminococcaceae*, together with small-intestinal injury, increased permeability, higher IFN-γ, IL-1β, and TNF-α, lower GLP-1R and GLP-2R expression, and enhanced lymphocyte migration to the intestinal mucosa through the MAdCAM-1-β7 integrin axis [68]. A liver-focused experiment also showed lower gut microbial α-diversity, reflected by a reduced ACE index, together with significant separation of gut microbial β-diversity. The microbial profile included lower abundances of *Faecalibacillus*, *Bifidobacterium*, *Akkermansia*, *Bacillus* and *Eggerthella* and higher abundances of *Collinsella*, *Caproiciproducens* and related taxa, while the corresponding host findings included elevated serum and hepatic long-chain fatty acids, reduced carnitine metabolites, lower β-oxidation, hepatic triglyceride accumulation, inflammatory cell infiltration, and higher circulating ALP, LPS, IL-6 and TNF-α [69]. Sex-dependent responses were also observed in healthy adult mice. Ace-K increased *Bacteroides*, *Anaerostipes* and *Sutterella* in males, but reduced *Lactobacillus*, *Clostridium* and unassigned *Ruminococcaceae* and increased *Mucispirillum* in females. These compositional differences were accompanied by shifts in microbial functional genes related to carbohydrate metabolism, fermentation, and LPS-associated processes, together with altered fecal metabolites, and increased body weight gain was detected only in males [22]. Direct microbial responsiveness has also been documented in mechanistic settings, including strong bacteriostatic activity against *E. coli* [41].

By contrast, several studies did not identify a strong or consistent Ace-K-associated gut microbiota signal. In a short ex vivo human gut microbiota platform, Ace-K did not measurably change gut microbial diversity, cell density, community composition, or metabolite production over 48 h [51], and a broader ex vivo metaproteomic screen likewise suggested only limited global perturbation within the target sweetener group [60]. In young adult rats, Ace-K was associated with only minor gut microbial changes and relatively limited plasma metabolomic differences, with the main signal involving conjugated bile acids rather than overt gut microbial disruption or a clear disease-related outcome [66]. Long-term comparative work in mice also did not identify a stable Ace-K-specific gut microbiota signature, and gut microbial α-diversity did not differ significantly between target sweetener groups and water controls, with the principal variation being explained by diet and exposure time rather than by Ace-K alone [14]. Similarly, early-life low-calorie sweetener exposure showed that Ace-K could impair postprandial glucose handling and alter behavior without robust gut microbiota changes, and the long-term outcome in that model was interpreted as not clearly gut microbiota-dependent [67].

#### 5.2.5. Neohesperidin Dihydrochalcone

Evidence on NHDC remains limited and is derived mainly from animal disease models and ex vivo systems. Available studies report potentially favorable microbiota-associated and metabolic signals under selected preclinical conditions, but their translational relevance remains uncertain. In high-fat-diet rats and diabetic zebrafish, NHDC shifted gut microbiota away from disease-associated profiles, including reversal of increased *Proteobacteria* and the *Firmicutes*/*Bacteroidetes* ratio (F/B ratio), enrichment of *Bifidobacterium*, *Clostridium*, *Oscillospira*, *Faecalibacterium* and related taxa, and higher acetate, propionate, or butyrate. These microbial changes were reported together with lower fasting glucose and insulin, improved lipid indices, reduced inflammatory cytokines, improved antioxidant status, better intestinal morphology and barrier-related markers, and attenuation of hepatic steatosis or glycolipid metabolism disturbance [70,71]. Ex vivo screening likewise did not indicate marked global disruption of the gut microbiome after NHDC exposure but did identify selected genus-level and metaproteomic changes [60]. An NHDC-containing combination with rebaudioside A (a natural sweetener) was also associated with reduced weight gain, lower adiposity, and improved hepatic lipid handling, together with higher *Blautia* and *Parabacteroides* and lower *Faecalibaculum* and *Mucispirillum* [72]. In studies where NHDC was examined alongside sucralose or saccharin, NHDC did not reproduce the *Akkermansia* depletion, bile acid disturbance, or NAFLD-like pattern reported for those sweeteners under parallel experimental conditions. In summary, the current evidence more often links NHDC with gut microbiota-associated improvement in glycolipid and intestinal outcomes than with gut microbiota-associated injury, although the number of available studies remains small. These findings should therefore be regarded as promising but currently preclinical, as the available evidence is derived predominantly from animal and ex vivo studies, with human data still lacking.

#### 5.2.6. Neotame

Neotame has so far been associated mainly with gut microbiota restructuring and fecal metabolite changes, whereas evidence directly linking these alterations to defined host metabolic or health outcomes remains limited. In mice, neotame reduced gut microbial α-diversity, produced clear separation in gut microbial β-diversity, increased the microbial dysbiosis index, enriched *Bacteroidetes* and S24-7-related taxa, and reduced several members of *Lachnospiraceae* and *Ruminococcaceae*, including *Blautia*, *Dorea*, *Oscillospira* and *Ruminococcus* [73]. These compositional changes were accompanied by lower predicted butyrate-forming capacity and by marked fecal metabolite shifts involving fatty acids, cholesterol-related compounds, and central carbon metabolism. However, this study did not show clear effects on body weight, eating behavior or general behavior. Broader ex vivo metaproteomic screening likewise did not indicate strong global disruption after neotame exposure, but rather limited microbiome perturbation with selected taxonomic and protein-level changes [60]. In adults with overweight or obesity, fruit-filled biscuits sweetened with neotame reduced the postprandial incremental area under the curve for insulin (insulin iAUC) compared with sucrose, whereas postprandial glucose showed only a decreasing trend without statistical significance and no significant differences were observed in composite appetite score, explicit liking, implicit wanting, ghrelin, GLP-1, or pancreatic polypeptide [74]. Current findings therefore support measurable neotame effects on gut microbial and fecal metabolite-related readouts in experimental systems, but they do not yet establish a consistent host metabolic phenotype, and human studies remain limited.

#### 5.2.7. Cyclamate

Cyclamate requires particularly cautious interpretation because gut microbiota-focused evidence remains sparse, and its broader safety and regulatory history should not be treated as proof of a gut microbiota-mediated effect. One in vitro study reported that cyclamate did not cause marked global disturbance of the gut microbiota, but was still associated with changes in the relative abundance of selected taxa and in microbial protein expression profiles [60]. A more recent animal study reported that subchronic sodium cyclamate exposure was associated with activation of inflammatory signaling, oxidative stress, apoptosis-related changes and overt cardiac tissue injury [75]. This toxicity signal is relevant to safety interpretation, but it does not by itself establish a gut microbiota mechanism. Current evidence is therefore insufficient to draw strong conclusions about cyclamate effects on gut microbiota, host metabolism or gut microbiota-linked health outcomes, and human microbiome studies remain a clear evidence gap.

#### 5.2.8. Mixed NAS

In addition to studies on individual NAS and commercial products predominantly formulated with a single NAS, some studies have examined the effects of mixtures containing multiple NASs. Mixed NAS exposures add an additional interpretive layer because they combine multiple compounds, commercial matrices, and dietary substitutions within the same intervention or observational exposure. In mixtures containing sucralose and Ace-K, the clearer gut microbiota-related signal was attributed mainly to sucralose, which reduced *Clostridium* cluster XIVa and lowered cecal butyrate, while the same combination was also associated with reduced gut microbial richness and increased jejunal glucose absorption [38,76]. Maternal exposure to sucralose and Ace-K further altered offspring gut microbiota, with higher *Firmicutes* and markedly lower *Akkermansia muciniphila*, together with broad fecal and plasma metabolomic disturbance and predicted later metabolic dysregulation [77]. In functional gastrointestinal disorders, a mixed NAS dietary pattern composed of approximately 80% sucralose and 20% aspartame, Ace-K and saccharin worsened diarrhea, postprandial discomfort, constipation, and retrosternal burning [78]. Long-term use of NAS packets or tablets was also associated with a higher risk of type 2 diabetes in a prospective cohort [79]. At the microbial functional level, mixtures containing saccharin, sucralose and aspartame increased biofilm formation, adhesion, and invasion-related behavior in model gut bacteria [53], while exposure to saccharin, sucralose, aspartame, and Ace-K increased ROS generation and plasmid-mediated conjugative transfer in fecal microbial communities, with the strongest effect observed for sucralose [55].

Not all mixed-exposure studies showed clear deterioration in gut microbiota or host metabolic and health-related outcomes. Diet sodas containing sucralose and Ace-K changed selected gut microbiota species, including increases in Gammaproteobacteria- and Enterobacteriaceae-related taxa after longer exposure, but did not significantly alter gut microbial α-diversity, gut microbial β-diversity, body weight, or energy intake [80]. In the SWEET study, mixed sweeteners and sweetness enhancers altered overall gut microbiota composition and supported one-year weight maintenance compared with sugar but did not produce major deterioration in cardiometabolic markers and did not generate a between-group difference in gut microbial α-diversity over time [13]. These findings indicate that mixed NAS exposures can be biologically active at the level of gut microbiota and host responses, but the direction and magnitude of effect depend on mixture composition, dominant sweetener, dietary matrix, comparator and host background. Interpretation should therefore combine evidence from single-sweetener and mixed-exposure studies without treating NASs as functionally uniform.

## 6. Interpreting Divergent Findings Across Studies

The current evidence does not support a single, uniform relationship between NAS exposure, gut microbiota changes, and host metabolic or health-related outcomes. Divergent findings are better interpreted as structured heterogeneity than as simple inconsistency. The major sources of heterogeneity include compound identity, dose, exposure duration, formulation, developmental window, host metabolic or inflammatory background, baseline gut microbiota, habitual NAS use, comparator, gut microbiota endpoint, host endpoint, measurement method, and the level of causal support. Within this framework, adverse, neutral, uncertain, and apparently favorable findings can coexist without implying that NAS act as a biologically uniform class. Table 2 presents a multidimensional evidence matrix designed to characterize structured heterogeneity across representative studies. Studies are organized by NAS type and form, exposure dose and duration, study type and research model, gut microbiota composition, microbial functional or metabolite endpoints, host phenotype, and causal validation. The study type, model, and causal validation fields support evidence-level comparison across human RCTs and cohorts, animal studies, ex vivo systems, defined communities, and single-strain assays. For evidence-rich categories, particularly sucralose, aspartame, saccharin, Ace-K, and mixed NAS exposures, studies with doses reported or normalized as mg/kg bw/day were prioritized where available to improve cross-study comparability. In vitro and ex vivo studies retained their original concentrations, while other informative studies retained original exposure metrics when body weight normalization was not applicable or could not be reliably reconstructed. Based on the literature-derived evidence records curated for this review, we developed NAS-MAP, a prototype knowledge graph and question-answering framework designed to organize relationships among NAS exposure, gut microbiota changes, and host metabolic or health-related outcomes. Table 2 is not the full NAS-MAP dataset. It is a compact evidence matrix showing the most comparable dose-reported studies in the main text.

### 6.1. Exposure Conditions

Variation in exposure conditions is one of the clearest explanations for cross-study divergence. Even for the same compound, the observed response can change with dose, duration, developmental window, and formulation. In mice exposed from weaning, low-dose sucralose reduced *Clostridium* cluster XIVa without a broad community shift or clear host phenotype, whereas a 10-fold higher dose over the same period produced the same taxon-level signal together with higher hepatic cholesterol [38]. Under longer exposure, the reported profile extended further, with altered gut microbial β-diversity, higher *Bacteroides* and *Clostridium*, elevated deoxycholic acid, hepatic lipid accumulation, and glucose intolerance [35]. A related study at the same nominal daily dose linked sucralose with lower *Akkermansia muciniphila*, increased gut permeability, systemic inflammation, and hepatic inflammation despite the absence of a significant body weight effect [36]. Six month exposure further connected microbiota changes with lower *Lachnospiraceae* and *Akkermansiaceae*, altered bile acid metabolism-related gene richness, hepatic cholesterol, and lipid accumulation [33]. These studies do not show that longer or higher exposure is automatically harmful. They show that dose and duration can shift the visible response from a restricted taxonomic change to functional and host-level outcomes.

Temporal context is another important modifier of the exposure–response relationship across NAS studies. Beyond developmental windows, circadian phase, feeding schedule, and the timing of glucose testing or microbiota sampling relative to NAS intake may influence observed responses. These timing variables may interact with host susceptibility and should therefore be considered when comparing metabolic and microbial outcomes across studies. Maternal sucralose exposure altered offspring gut microbiota at weaning, including lower α-diversity, altered β-diversity, changes in *Akkermansia* and other taxa, and impaired intestinal development, barrier function, and inflammatory status. When offspring were later challenged with a high-fat diet, the dominant phenotype shifted toward greater body weight gain, higher liver weight, increased adiposity, and hepatic inflammation [39]. This pattern indicates that exposure timing and subsequent metabolic challenge help determine which layer of the NAS–gut microbiota–host metabolism axis becomes visible. Formulation is another source of heterogeneity. Product-based exposures cannot be assumed to reproduce purified compound effects because commercial preparations and mixed sweetener systems introduce co-formulated ingredients, unequal compound contributions, and diet-dependent matrix effects [9,42,43].

Dose comparisons also remain limited when studies report intake as drinking water concentration, dietary percentage, packets, tablets, beverages, or maternal exposure rather than as a standardized daily intake. Nominal mg/kg bw/day values should therefore be read together with the original exposure matrix, conversion assumptions, species or model background, and regulatory dose context. Exposure conditions are not a peripheral design detail. They are part of the biological result and should be interpreted before cross-study conclusions are drawn.

### 6.2. Research Model Background

Research model background is another major determinant of divergent findings. The same NAS exposure may produce different patterns in healthy hosts, disease-prone hosts, maternal exposure models, or human participants with different baseline gut microbiota and metabolic states. Aspartame provides a clear example. Under normal physiological conditions, aspartame altered gut microbiota structure without overt host pathology, whereas the same dose and exposure duration in a DSS-induced colitis model were accompanied by more severe intestinal injury, higher inflammatory cytokine levels, and greater epithelial barrier damage [61]. NHDC shows the opposite direction in selected disease contexts. It did not significantly alter gut microbiota or host outcomes in normal mice, but in high-fat-diet or diabetic models it was associated with gut microbial restructuring and lower fasting glucose, fasting insulin, and serum triglycerides [36,70,71]. These comparisons indicate that the biological meaning of a sweetener exposure is not determined only by the compound itself. It is also shaped by the metabolic or inflammatory background in which that exposure is tested.

Human and animal evidence should therefore be compared by model role rather than by apparent outcome direction alone. Short-term sucralose exposure in healthy men did not significantly alter microbiota composition, glycemic control, or insulin resistance [52], whereas longer exposure in healthy lean adults with low habitual non-nutritive sweetener intake was associated with lower gut microbial α-diversity, altered β-diversity, higher abundances of *Bacteroides* and related taxa, and poorer glucose and insulin responses [11]. Saccharin also demonstrates response heterogeneity within humans. Responders developed microbiota changes together with poorer glycemic responses, whereas non-responders did not show the same pattern [9]. These findings suggest that baseline gut microbiota, habitual exposure history, and host susceptibility should be treated as part of the response rather than as background noise.

Different experimental systems also capture different inference layers. Direct microbial assays and ex vivo communities can identify growth, adhesion, quorum-sensing, SCFA, plasmid transfer, or fermentation effects [53,54,55], but they do not capture the host setting in which barrier injury, inflammatory tone, liver metabolism, or endocrine regulation become part of the response. Conversely, integrated animal and human studies may detect host metabolic and health-related outcomes even when broad gut microbiota restructuring is limited. Ace-K illustrates this point: impaired glucose handling and behavioral change were reported in early-life exposure settings without robust gut microbiota alteration [67], whereas other Ace-K studies showed clear gut microbiota changes together with intestinal inflammation or hepatic metabolic injury [68,69]. Maternal exposure models add a further layer because the relevant outcome may emerge in offspring rather than in the exposed mother, as shown for sucralose and aspartame [39,56,58]. Overall, these findings show that research model background influences not only whether an effect is observed, but also which component of the gut microbiota–host relationship becomes most visible. For this reason, differences across models should be interpreted as biologically informative rather than being treated as purely methodological variation.

### 6.3. Outcome Assessment

A further source of divergence is that studies do not measure the same response layer. Some focus on the community composition of the gut microbiota, others on gut microbial metabolites or functional pathways, and others on host metabolic, inflammatory, or barrier outcomes. These layers do not necessarily change in parallel. In sucralose-exposed mice, a low dose reduced *Clostridium* cluster XIVa and cecal butyrate without broad community restructuring or clear host outcomes, whereas longer or more intensive exposure aligned with altered gut microbial β-diversity, bile acid disturbance, hepatic lipid accumulation, glucose intolerance, or gut barrier impairment [35,36,38]. Neotame likewise altered gut microbial α-diversity, β-diversity, *Lachnospiraceae* and *Ruminococcaceae*-related taxa, and fecal metabolites, but did not produce clear body weight or general behavioral changes within the same observation window [73]. These findings indicate that gut microbiota composition can shift before overt host metabolic and health-related outcomes become clearly detectable.

The reverse pattern is also possible. A host phenotype may be measurable even when broad gut microbiota changes are weak or absent. Early-life Ace-K exposure impaired postprandial glucose handling and altered behavior, but robust gut microbiota changes were not detected and the long-term phenotype was not clearly gut microbiota-dependent [67]. In young adult rats, Ace-K was accompanied mainly by limited gut microbiota change and conjugated bile acid-related metabolite differences rather than by a strong community-wide signal [66]. Human studies point in the same direction. Short-term pure aspartame or sucralose interventions in healthy adults left microbiota composition and glycemic control largely unchanged under the tested conditions [25,52], whereas other human studies reported altered glycemic responses, circulating inflammatory markers, or gastrointestinal symptoms together with selective gut microbial or species-level changes rather than a uniform shift in diversity indices [11,46,59,78,80]. The implication is important for interpretation. The absence of a broad diversity signal does not prove biological inactivity. A detectable gut microbiota change does not by itself prove a defined host metabolic consequence.

This uneven alignment across response layers also affects how different sweeteners are compared. Saccharin and sucralose more often appear in studies where gut microbiota alteration is discussed together with glucose intolerance or liver-related outcomes [9,10,33,35,36], whereas preclinical NHDC studies in high-fat-diet or diabetic models reported gut microbial restructuring alongside improvements in selected metabolic indices [70,71]. Ace-K, by contrast, often shows a stronger signal at the level of intestinal injury, hepatic lipid disturbance, or inflammatory markers than at the level of a single reproducible gut microbiota signature [66,67,68,69]. These differences indicate that divergence across studies is not explained only by whether an effect is present or absent. It is also shaped by which part of the gut microbiota–host axis is captured by the study design and treated as the primary endpoint.

### 6.4. Interpreting Discordant Results

Discordant results should not be reduced to a contrast between positive and negative studies. A more useful question is whether each result can be placed within a coherent context of exposure, host background, endpoint layer, and causal support. The sucralose literature illustrates this logic. Some studies associate sucralose with lower *Faecalibacterium prausnitzii*, *Lactobacillus*, or *Akkermansia muciniphila*, altered bile acid metabolism, increased gut permeability, hepatic inflammation, and impaired glycemic control [11,33,35,36,39,46]. Other studies report no significant changes in community composition, fecal SCFAs, glucose metabolism, or insulin sensitivity under short-term or controlled pure compound exposure [25,51,52]. These observations do not cancel each other out. They define the contexts in which sucralose-associated responses were or were not detected. The same logic applies to saccharin, where responder-dependent glycemic effects and high-dose null findings in healthy models should be interpreted as context-bounded evidence rather than as mutually exclusive verdicts.

This point becomes even clearer when studies reporting apparently favorable outcomes are also considered. NHDC did not significantly alter gut microbiota composition or host metabolic and health-related outcomes in normal mice [36], but under high-fat-diet or diabetic conditions it was associated with lower fasting glucose, insulin, and triglycerides, improved intestinal or hepatic indices, and microbial changes including lower *Proteobacteria* or a reduced F/B ratio [70,71]. Such findings should not be treated as exceptions outside the main argument. They show that NAS-associated gut microbial changes can align with adverse, neutral, or favorable host readouts depending on compound identity and host context. Responder-dependent human data support the same point. In saccharin-exposed healthy volunteers, only responders developed gut microbiota alteration together with poorer glycemic responses, whereas non-responders did not show the same pattern [9]. Divergence is therefore evidence that host susceptibility and baseline gut microbial configuration matter.

For this reason, studies showing adverse effects, limited responses, and no significant effect should be interpreted within the same framework. Null findings are not failed results. They define the conditions under which a given response was not detected and therefore help delimit the scope of broader claims. Positive findings, meanwhile, need to be read in relation to the exact context in which they were generated, rather than being generalized to all sweeteners or all exposure settings. The present literature therefore does not justify a single overall statement that NAS either disrupt or do not disrupt gut microbiota and host metabolic and health-related outcomes. What it does justify is a more precise conclusion: different sweeteners generate different response profiles, and those profiles are shaped by exposure conditions, research model background, the level at which responses are assessed, and the coexistence of adverse, neutral and, in some cases, apparently favorable outcomes within the same evidence base.

### 6.5. NAS-MAP Knowledge Graph as a Lightweight Evidence Organization Framework

**Figure 5:**
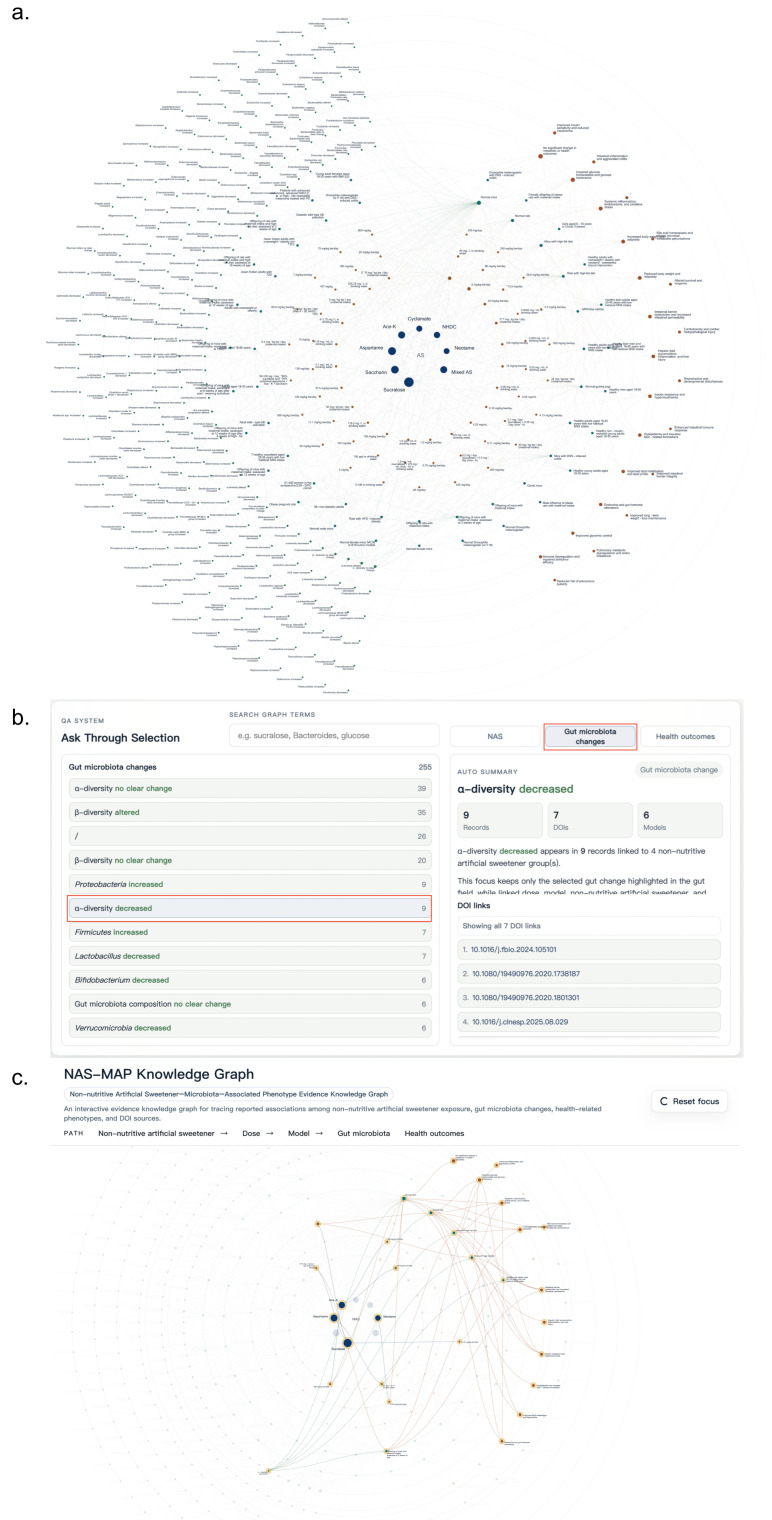
NAS-MAP knowledge graph interface for mapping NAS–gut microbiota–host metabolism relationships. (**a**) Global radial evidence graph linking NAS categories, dose settings, research models, gut microbiota changes, and health-related phenotypes; (**b**) selection-based QA panel using ‘α-diversity decreased’ as an example, showing linked evidence records, model contexts, DOI-linked articles, and source links; (**c**) focused graph view after selecting ‘α-diversity decreased’, with connected evidence paths highlighted and unrelated nodes faded.

To make the heterogeneous evidence base more traceable, we developed the NAS-MAP knowledge graph (http://www.nasmap.net/), an interactive non-nutritive artificial sweetener–microbiota-associated phenotype knowledge graph and selection-based question-answering (QA) interface. The framework connects individual NASs and mixed exposure with reported dose settings, research models, gut microbiota-related findings, host phenotypes, and DOI-linked sources. Its global view presents these elements as connected evidence paths, allowing users to examine how specific microbial or host outcomes were reported across different compounds and experimental contexts (Figure 5a).

Below the graph, the QA panel allows users to search or select terms from ‘NAS’, gut ‘microbiota changes’, and ‘health outcomes’, and then inspect the automatically generated summary and DOI links for the selected focus. This design turns the graph from a passive visualization into a small evidence-questioning system. For example, when a user selects ‘α-diversity decreased’ in the ‘gut microbiota changes’ panel, the right-hand summary reports nine records, seven DOI-linked articles, and six model contexts, with clickable DOI links for source verification (Figure 5b). The same panel also provides a concise textual interpretation rather than merely displaying the selected nodes. In the ‘α-diversity decreased’ example, it indicates that this endpoint appears in nine records across four NAS groups and keeps the selected gut microbiota change visually connected to its linked dose, model, NAS, and health outcome nodes. The co-reported host outcomes cover both neutral and adverse findings, including no significant metabolic or health-related changes, impaired glucose homeostasis, systemic inflammation or oxidative stress, increased body weight and adiposity, and intestinal barrier dysfunction. This example illustrates why the graph should be interpreted as an evidence map rather than as a causal scoring system: the same gut microbiota endpoint may be reported together with neutral, adverse, or context-dependent host outcomes across different sweeteners and models. After selection, the graph updates to retain the ‘α-diversity decreased’ node and highlight its connected NAS, dose, model, and phenotype paths while fading unrelated background nodes, showing how the selected endpoint is distributed across sucralose, aspartame, Ace-K, neotame, and their experimental contexts (Figure 5c). NAS-MAP is therefore intended as a team-managed framework rather than a comprehensive database. It represents the first lightweight interactive evidence organization framework designed specifically for NAS–gut microbiota–host metabolism relationships, but its current value lies primarily in traceability and structured synthesis rather than in database scale or quantitative evidence grading. However, its limitations are also instructive. The current data volume remains limited, evidence strength and effect sizes are not yet graded, and the graph itself exposes the field’s lack of standardized models, dose metrics, outcome definitions, and machine-readable reporting. Future extensions should therefore prioritize standardized, quantitative, and interoperable evidence fields so that machine learning, deep learning, and other AI models can be applied more robustly to this research area.

## 7. Challenges and Future Prospects

### 7.1. Limitations and Challenges

Exposure measurement remains a central limitation of the NAS literature. Studies report intake as mg/kg body weight per day, concentrations in drinking water or feed, product servings, or percentages of acceptable daily intake. These metrics describe different exposure matrices and cannot be treated as biologically interchangeable, even after nominal dose conversion. Human studies add further uncertainty because food frequency questionnaires and 24 h recalls are vulnerable to recall error, underreporting, and incomplete identification of individual compounds within commercial mixtures. Food composition databases rarely provide sufficient formulation detail to recover compound-specific intake reliably [83]. Consequently, current evidence cannot yet support robust cross-study dose–response synthesis or fully harmonized, machine-readable exposure records.

Translational interpretation is also constrained by a mismatch between commonly studied populations and those most likely to seek guidance on NAS use. Many interventions enroll healthy, normoglycemic participants, whereas habitual users may include individuals with obesity, metabolic syndrome, prediabetes, or type 2 diabetes. Responses observed in healthy participants cannot be assumed to apply to metabolically compromised populations. These populations may differ in intestinal barrier function, inflammatory tone, bile acid metabolism, and microbial ecology [84]. Evidence from human interventions, animal disease models, and in vitro or ex vivo systems should therefore be interpreted according to the distinct questions that each design can answer. The limited representation of clinically relevant populations restricts the translation of mechanistic observations into dietary guidance.

Gut microbiota assessment frequently remains too coarse to distinguish association from mechanism. Many studies rely on relative taxonomic abundance, α-diversity, and β-diversity, which can identify community shifts but cannot determine whether those shifts alter microbial function or mediate host outcomes. Emerging work suggests that inter-species interaction architecture may provide information beyond compositional inventories, although its value as an NAS-specific biomarker remains to be established [85]. Mechanistic validation using gnotobiotic models, fecal microbiota transplantation, targeted metabolite tracing, perturbation experiments, and integrated multi-omics analyses remains uncommon [86]. Accordingly, an unchanged diversity metric does not exclude functional or host-level effects, and a detectable microbiota shift does not by itself establish a clinically meaningful consequence.

### 7.2. Future Prospects

The limitations reviewed above collectively point toward research directions that are not merely incremental refinements but represent genuine paradigm-level reorganizations of how this field must be pursued. Future studies should move beyond taxonomic cataloging toward designs that connect ecological structure, gut microbial function, and host response. Interaction network metrics may help determine whether a compound alters community organization rather than only relative abundance, but these metrics require validation within NAS-specific interventions [85]. Their interpretation should be integrated with metagenomics, metatranscriptomics, metabolomics, and direct perturbation experiments. Such designs could distinguish reproducible gut microbial mediation from context-dependent association and improve the mechanistic resolution of the field [84,86].

Progress also depends on more precise and interoperable exposure measurement. Objective biomarkers, time-stamped dietary records, image-assisted assessment, and complementary molecular approaches may reduce reliance on self-report. Each method, however, requires validation for specific compounds and commercial formulations [83]. This shift matters because it enables the data infrastructure on which AI-driven precision nutrition depends. Progress in machine learning-based glycemic response prediction has shown that incorporating gut microbiota features meaningfully improves model performance beyond conventional clinical variables in metabolically complex populations [87], and multi-omics phenotyping has established that metabolome metrics linked to microbial activity capture cardiometabolic risk with substantially greater precision than anthropometric surrogates alone [88]. However, algorithmic performance cannot compensate for inconsistent exposure definitions, poorly aligned endpoints, or non-comparable study designs. Standardized, compound-resolved, and machine-readable reporting is therefore a prerequisite for responsible computational integration [89]. Within this agenda, NAS-MAP should evolve through collaboration rather than remain a resource curated solely by one research team. The current version is a manually organized knowledge-based prototype that demonstrates how heterogeneous evidence can be made traceable. Developing it into a quantitative resource that can inform regulatory assessment will require multi-team curation and participation from nutrition scientists, microbiome researchers, analytical chemists, clinicians, data curators, and regulatory experts. Shared ontologies, transparent inclusion criteria, versioned provenance, harmonized dose fields, and explicit effect size and certainty metadata will be essential. Regulatory translation must depend on independent validation and engagement with relevant authorities rather than on the framework alone. Most importantly, prospective studies using standardized experimental designs must generate reliable, comparable, and health-relevant evidence that can populate and test the framework.

Future intervention studies should therefore pre-specify circadian phase, exposure timing, habitual NAS use, baseline microbiome features, metabolic status, sex, formulation, and comparator. Repeated sampling and time-stamped intake records would help determine whether an apparent response is transient, cumulative, or dependent on the host’s metabolic and circadian context [90]. Studies should therefore pre-specify exposure timing, habitual NAS use, baseline microbiome features, metabolic status, sex, formulation, and comparator. Repeated sampling and time-stamped intake records would help determine whether an apparent response is transient, cumulative, or dependent on the host’s metabolic and circadian context.

Candidate sweeteners should be evaluated comparatively rather than presumed safer because they are naturally derived. Future studies should compare synthetic, semi-synthetic, and naturally derived high-intensity sweeteners under matched dose units, exposure matrices, host backgrounds, microbiome readouts, and metabolic endpoints. Scalable production methods support further investigation of Reb D and Reb M [91]. Preclinical research in high-fat diet models has shown that neither compound exacerbates obesity-associated phenotypes including body weight gain, hepatic steatosis, or skeletal muscle metabolic impairment, and that prolonged exposure is associated with favorable microbial reorganization toward beneficial taxa relative to caloric sugar comparators [92]. However, current evidence remains insufficient to establish superior long-term human health or microbiome outcomes. Their value in this research agenda lies in enabling rigorous comparison, not in serving as predetermined replacements for synthetic NAS.

## 8. Conclusions

Current evidence supports a conditional, compound-specific account of how NASs interact with the gut microbiota and host physiology. NASs are chemically distinct exposures whose biological effects depend on dose, duration, formulation, developmental timing, habitual use, and the host’s metabolic or inflammatory state. Microbial mediation is the most credible when microbial changes align with functional readouts and host phenotypes and when perturbation or transfer experiments reproduce the relationship. In vitro and ex vivo systems identify possible interactions, while animal models test biological plausibility and host susceptibility. Human interventions assess whether such effects are detectable under defined exposure conditions in people. These evidence layers show why apparently divergent findings can coexist. Sucralose and saccharin have the most developed evidence for context-dependent gut microbiota-linked metabolic or barrier effects. Nevertheless, controlled human and experimental studies have reported limited or null responses. Findings for aspartame and Ace-K are similarly heterogeneous. NHDC has shown promising microbiota-associated signals, but the evidence remains preclinical and is derived mainly from animal disease models and ex vivo systems. Evidence for neotame and cyclamate remains insufficient for strong gut microbiota-related conclusions. Accordingly, a compositional shift should not be equated with functional disruption, gut microbial mediation, or clinical harm. Conversely, an unchanged diversity metric should not be taken as evidence of biological inactivity.

The principal limitation is therefore not inconsistency itself, but the poor comparability of exposures, populations, models, and endpoints used to generate the evidence. Progress requires compound-resolved and formulation-aware exposure assessment, realistic dose reporting, clinically relevant populations, repeated sampling, and standardized outcomes that connect gut microbial ecology and function to host physiology. These designs should be integrated with multi-omics profiling and targeted mechanistic validation, while preserving uncertainty where causal support is absent.

By linking field-level trends with compound-resolved evidence across experimental scales, this review provides a structured framework for distinguishing reproducible biological signals from context-dependent observations and for identifying where causal inference remains insufficient. The accompanying NAS-MAP knowledge graph further makes this heterogeneous evidence base traceable by connecting individual NAS, gut microbial alterations, host phenotypes, and experimental contexts within a unified structure. Such an evidence structure can support dietary and public health guidance specific to the sweetener, exposure context, and population. Neither adverse nor reassuring findings should be extended beyond the conditions in which they were observed.

## Figures and Tables

**Table 1:** Physicochemical characteristics and regulatory information of the major NASs (included in this review).

Sweetener	Formula	MW (g/mol)	Key Physicochemical Characteristics	Relative Sweetness vs. Sucrose	Regulatory Status	ADI (mg/kg bw/Day)
Ace-K (E950)	C_4_H_4_KNO_4_S	201.24	Highly water-soluble. Heat-stable.	200×	FDA: approved. EFSA: authorized as E950; re-evaluated in 2025. JECFA: evaluated.	FDA: 15 EFSA: 15 JECFA: 0–15
Aspartame (E951)	C_14_H_18_N_2_O_5_	294.30	Limited suitability under prolonged heating.	200×	FDA: approved. EFSA: authorized as E951. JECFA: evaluated; reaffirmed in 2023.	FDA: 50 EFSA: 40 JECFA: 40
Cyclamate (E952)	C_6_H_13_NO_3_S	179.24	Lower sweetness potency. Water-soluble salt forms.	30–40×	FDA: not approved. EFSA: authorized as E952; re-evaluation ongoing. JECFA: evaluated.	FDA: / EFSA: 7 (Re-evaluating) JECFA: 11
Saccharin (E954)	C_7_H_5_NO_3_S	183.19	Parent compound is sparingly soluble. Sodium salts are highly soluble.	200–700×	FDA: approved. EFSA: authorized as E954; re-evaluated (2024). JECFA: evaluated.	FDA: 15 EFSA: 9 JECFA: 5
Sucralose (E955)	C_12_H_19_C_l3_O_8_	397.64	Water-soluble.	600×	FDA: approved. EFSA: authorized as E955; re-evaluated in 2026. JECFA: evaluated.	FDA: 5 EFSA: 15 JECFA: 15
NHDC (E959)	C_28_H_36_O_15_	612.58	High sweetness potency. Produced by hydrogenation of neohesperidin from bitter orange.	950–1800×	FDA: not approved; GRAS Notice GRN 902 received a no-questions response. EFSA: authorized as E959; re-evaluated in 2022. JECFA: /	FDA: / EFSA: 20 JECFA: /
Neotame (E961)	C_20_H_30_N_2_O_5_	378.46	Extremely high sweetness potency. Practical exposure levels are generally low.	7000–13,000×	FDA: approved. EFSA: authorized as E961; re-evaluated in 2025. JECFA: evaluated.	FDA: 0.3 EFSA: 10 JECFA: 2

Abbreviations used: Ace-K, acesulfame potassium; ADI, acceptable daily intake; bw, body weight; EFSA, European Food Safety Authority; FDA, U.S. Food and Drug Administration; GRAS, Generally Recognized as Safe; GRN, GRAS Notice; JECFA, Joint FAO/WHO Expert Committee on Food Additives; MW, molecular weight; NHDC, neohesperidin dihydrochalcone; vs. = versus/compared with.

**Table 2:** Representative NAS studies with reported doses and paired microbiota and host-related outcomes.

Types of NAS	Form	Dose	Study Type	Research Model	Exposure Duration	Effects on Gut Microbiota Composition	Microbial Functional/Metabolite Endpoint Changes	Host Phenotype	Causal Validation	Ref.
Sucralose	Pure	1.5 mg/mL	Ex vivo	Healthy human fecal microbiota	24 h	Limited donor-specific taxonomic responses	Limited global metaproteomic change	/	/	[60]
Sucralose	Pure	1.67 mg/mL	Ex vivo	Healthy and IBD human fecal microbiota	72 h	*Roseburia* ↓; *Faecalibacterium prausnitzii* ↓; *Enterococcus* ↑; *Veillonella* ↑; *Mucispirillum schaedleri* ↑	SCFA profile altered	/	/	[31]
Sucralose	Pure	1.05 g/day	Ex vivo	Healthy and T2D human fecal microbiota	48 h	No significant change in diversity or community composition	No significant change in metabolite production	/	/	[51]
Sucralose	Pure	50% ADI	Ex vivo	Healthy human fecal microbiota	24 h	β-diversity altered; *Escherichia*/*Shigella* ↑; *Bilophila* ↑	Valerate ↑	/	/	[32]
Sucralose	Pure	5 mg/kg bw/day	Animal	Normal mice	6 months	*Lactobacillus* ↓; *Ruminococcus* ↓	Bile salt hydrolase gene richness ↓; secondary bile acid synthesis ↓	Hepatic cholesterol ↑; hepatic lipid accumulation ↑	/	[33]
Sucralose	Pure	60.6 mg/kg bw/day (chow); 28.6 mg/kg bw/day (HFD)	Animal	Normal and HFD mice	16 weeks	No significant change in bacterial load or Firmicutes/Bacteroidetes ratio	/	HFD: body weight gain ↓; insulin sensitivity ↑; hepatic steatosis ↓	/	[81]
Sucralose	Pure	1.5 or 15 mg/kg bw/day	Animal	Normal mice	8 weeks	*Clostridium cluster XIVa* ↓; no significant broad community change	Cecal butyrate ↓ (dose-dependent)	Hepatic cholesterol ↑ at 15 mg/kg bw/day	/	[38]
Sucralose	Pure	5 mg/kg bw/day	Animal	Normal mice	6 months	*Akkermansia*, *Ruminococcus*, *Anaerostipes* and *Roseburia* altered over time	LPS/flagellar/toxin genes ↑; quorum-sensing AHLs ↓; tryptophan and bile acid metabolites altered	Hepatic MMP-2 ↑; hepatic iNOS ↑	/	[21]
Sucralose	Pure	5 mg/kg bw/day	Animal	Normal mice	11 weeks	No significant change in α-diversity; β-diversity altered; *Bacteroides* ↑; *Clostridium* ↑	DCA ↑; total bile acids ↑	Hepatic lipid accumulation ↑; glucose intolerance ↑	Metformin/FOS rescue partially reversed the microbiota–DCA–FXR phenotype	[35]
Sucralose	Commercial product	5 mg/kg bw/day	Animal	Normal and HFD rats	8 weeks	No significant change in α-diversity, β-diversity or phylum-level composition; *Romboutsia* ↓ (normal diet); *Lactobacillus* ↓ (*HFD*)	/	/	/	[43]
Sucralose	Pure	5 mg/kg bw/day	Animal	Normal mice	11 weeks	No significant change in α-diversity; β-diversity altered; *Akkermansia muciniphila* ↓; *Lactobacillus* ↓; *Proteobacteria* ↑; *Streptococcus* ↑; *Prevotella* ↑	LPS biosynthesis genes ↑; propionate ↓; butyrate ↓; AhR ligands ↓	Gut permeability ↑; systemic/hepatic inflammation ↑; hepatic steatosis ↑	Metformin/FOS rescue restored *Akkermansia*/*AhR-ligand signaling and improved NAFLD*	[36]
Sucralose	Pure	5 mg/kg bw/day	Animal	Maternal-exposure mice/offspring	Gestation and lactation	α-diversity ↓; β-diversity altered; *Parabacteroides* ↑; *Akkermansia* ↑; *Blautia* ↑; *Bacteroides* ↓; *Clostridium XIVa* ↓	/	Offspring weight gain ↑; intestinal development impaired; Paneth cells ↓; intestinal inflammation ↑	/	[82]
Sucralose	Pure	5 mg/kg bw/day	Animal	Maternal-exposure mice/offspring + HFD challenge	Gestation/lactation + 4-week HFD challenge	α-diversity ↓ at weaning; β-diversity altered; butyrate-producing taxa ↓; Proteobacteria ↑; *Blautia* ↑; *Escherichia*/*Shigella* ↑	Cecal butyrate ↓; colonic GPR43 ↓	Gut barrier function ↓; adult adiposity ↑; hepatic steatosis/inflammation ↑	*Clostridium butyricum* rescue with GPR43 blockade (in vitro)	[39]
Sucralose	Pure	6.3–6.4 mg/kg bw/day	Human cohort + animal + cell model	Pregnancy cohort; maternal-exposure mice/offspring; adipocytes	Pregnancy and lactation	/	/	Offspring body weight/adiposity ↑; insulin sensitivity ↓; adipocyte lipid accumulation ↑	/	[30]
Sucralose	Pure	>0.16 mg/kg bw/day (human); ~0.45 mg/day (mouse)	Human cohort + animal	ICI-treated patients; tumor-bearing mice	Pretreatment + ICI course	Gut microbiota disrupted	Microbiota-accessible arginine ↓	Anti-PD-1 response ↓; tumor burden ↑; T-cell exhaustion ↑	Antibiotic depletion, cohousing and FMT; responder microbiota/amino acid rescue	[48]
Sucralose	Pure	75–300 mg/kg bw/day	Animal	Growing rabbits	8 weeks	Total bacteria ↑; *Lactobacillus* ↑; *Clostridium* spp. ↑; *Escherichia coli* ↓	Cecal ammonia ↓	Feed intake/body weight gain ↓ at 150–300 mg/kg; glucose ↓; triglycerides ↓; cholesterol/LDL ↑	/	[49]
Sucralose	Pure	5 mg/kg bw/day	Animal	Normal female mice	12 weeks	/	Serum LPS ↑	Insulin resistance ↑; ovarian follicular dysplasia ↑; reproductive hormones altered	Neomycin depletion reversed LPS/IL-6, insulin resistance and ovarian abnormalities	[45]
Sucralose	Pure	4.13 mg/kg bw/day	Human RCT	Healthy lean adults with low habitual NNS intake	30 days	α-diversity ↓; *Bacteroides fragilis* ↑; *Faecalibacterium prausnitzii* ↓; *Gram-negative bacteria* ↑	Acetate ↑; butyrate ↓; Curli protein ↑	Glucose/insulin/C-peptide/GLP-1 iAUC ↑; HOMA-IR ↑; insulin sensitivity ↓; inflammation ↑	/	[11]
Aspartame	Pure	2 mg/mL	Ex vivo	Healthy human fecal microbiota	24 h	Limited donor-specific taxonomic responses	Limited global metaproteomic change	/	/	[60]
Aspartame	Commercial product	500 mg	Ex vivo	Healthy human fecal microbiota	24 h	*Bifidobacterium* ↑	Acetate ↑; propionate ↑; branched-chain fatty acids ↓	/	/	[32]
Aspartame	Pure	5–7 mg/kg bw/day	Animal	Maternal HFS-obese rats and offspring	Gestation and lactation	Offspring β-diversity altered; *Akkermansia muciniphila* ↑; *Limosilactobacillus reuteri* ↓; *Ligilactobacillus murinus* ↓	Propionate/butyrate pathways ↑; lactate production capacity ↓	Offspring weight gain ↑; body fat ↑; liver weight ↑; bone mineral density ↓	/	[56]
Aspartame	Pure	40 mg/kg bw/day	Animal	Normal and DSS-colitis mice	7 days	α-diversity ↑; β-diversity altered; *Lactobacillus vaginalis* ↑; *Lactobacillus intestinalis* ↑; *Alloprevotella* ↑; *Parabacteroides* ↑; *Adlercreutzia mucosicola* ↓	/	No significant gut pathology in normal mice; colitis severity ↑; barrier injury ↑; systemic inflammation ↑	/	[61]
Aspartame	Pure	30 or 60 mg/kg bw/day (animal); habitual intake (human)	Animal + human cohort	Maternal-exposure rats/offspring; girls cohort	To puberty (animal); 3-month dietary (human)	Animal β-diversity altered; *Romboutsia* ↑; *Escherichia*/*Shigella* ↑; no significant diversity change in humans	Fecal SCFAs ↓	Pubertal onset delayed; estrogen ↓; LH ↑	/	[29]
Aspartame	Pure	5–7 mg/kg bw/day	Animal	Normal and HFD rats	8 weeks	Enterobacteriaceae ↑; *Clostridium leptum* ↑; *Roseburia* ↑ (*HFD*); *Firmicutes*/*Bacteroidetes ratio* ↓	Circulating propionate ↑; acetate/butyrate ↑ (normal diet)	Body fat ↓; fasting glucose ↑; insulin tolerance ↓; plasma free fatty acids ↑	/	[20]
Aspartame	Pure	40 mg/kg bw/day	Animal	Maternal-exposure mice/offspring	Gestational day 7 to postnatal day 21	/	Host–microbiota co-metabolic pathways altered	Pulmonary uric acid ↑; hypoxanthine ↓; oxidative stress ↑; inflammasome activation ↑	/	[62]
Aspartame	Pure	5–7 mg/kg bw/day	Animal	Maternal HFS-obese rats/offspring	Gestation and lactation	*Clostridium leptum* ↑	Cecal isovalerate ↑; valerate ↑	Offspring adiposity ↑; glucose tolerance ↓; insulin sensitivity ↓	Offspring cecal FMT transferred adiposity and impaired glucose tolerance to germ-free mice	[58]
Aspartame	Pure	32–42 mg/kg bw/day	Human cohort + animal + cell model	Pregnancy cohort; maternal-exposure mice/offspring; adipocytes	Pregnancy and lactation	/	/	Offspring body weight/adiposity ↑; insulin sensitivity ↓	/	[30]
Saccharin	Pure	0.2 mg/mL	Defined microbial community	SIHUMIx eight-species community	24 h	*Clostridium butyricum* ↓ ~75%; *Escherichia coli* ↑ ~2.5-fold	SCFAs and metabolic pathways altered	/	/	[63]
Saccharin	Commercial + pure	5 mg/kg bw/day	Human cohort + human intervention + animal	Healthy adults; normal/HFD mice; germ-free recipients	7 days (human); 5–11 weeks (mice)	*Bacteroides* ↑; *selected Clostridiales altered*; *Lactobacillus reuteri* ↓; *Akkermansia muciniphila* ↓	Glycan degradation pathways ↑; acetate ↑; propionate ↑	Glucose intolerance ↑	Antibiotic abrogation; FMT and conditioned microbiota transfer to germ-free mice	[9]
Saccharin	Pure	15 mg/kg bw/day	Animal	Normal juvenile rats	P26–P60	No significant change in α-diversity, β-diversity or taxonomic composition	/	Postprandial glucose handling impaired; memory ↓; sugar-motivated behavior altered	/	[67]
Saccharin	Pure	5 mg/kg bw/day (human); 250 mg/kg bw/day (mouse)	Human RCT + animal	Healthy adults; normal WT/T1R2-KO mice	2 weeks (human); 10 weeks (mice)	No significant change in α-diversity, β-diversity or taxonomic composition	No significant change in fecal SCFAs or metabolome	No significant change in body weight, glucose tolerance, insulin, GLP-1 or intestinal permeability	/	[26]
Saccharin	Pure	5 mg/kg bw/day	Animal	Normal mice	11 weeks	No significant change in α-diversity; β-diversity altered; *Akkermansia muciniphila* ↓; *Lactobacillus* ↓; *Proteobacteria* ↑; *Streptococcus* ↑; *Prevotella* ↑	LPS biosynthesis genes ↑; propionate ↓; butyrate ↓; AhR ligands ↓	Gut permeability ↑; systemic/hepatic inflammation ↑; hepatic steatosis ↑	Metformin/FOS rescue restored *Akkermansia*/*AhR-ligand signaling and improved NAFLD*	[36]
Ace-K	Pure	150 mg/kg bw/day	Animal	Normal mice	8 weeks	α-diversity ↓; β-diversity altered; Erysipelotrichaceae ↑; Clostridiaceae ↓; Lachnospiraceae ↓; Ruminococcaceae ↓	/	Small-intestinal injury ↑; permeability ↑; intestinal inflammation ↑	FMT did not reproduce intestinal injury	[68]
Ace-K	Pure	40 or 120 mg/kg bw every 2 days	Animal	Normal mice	11 weeks	α-diversity ↓; β-diversity altered; *Faecalibacillus* ↓; *Bifidobacterium* ↓; *Akkermansia* ↓; *Collinsella* ↑; *Caproiciproducens* ↑	Long-chain fatty acids ↑; carnitine metabolites ↓; β-oxidation ↓	hepatic triglycerides ↑; hepatic lipid accumulation ↑; systemic/hepatic inflammation ↑	/	[69]
Ace-K	Pure	15 mg/kg bw/day	Animal	Normal juvenile rats	P26–P60	No significant change in α-diversity, β-diversity or taxonomic composition	/	Postprandial glucose handling impaired; memory ↓; sugar-motivated behavior altered	/	[67]
Ace-K	Pure	15 mg/kg bw/day	Animal	Normal mice	8 weeks	No significant change in α-diversity or β-diversity; *Corynebacterium* ↓	/	No significant change in body weight, adiposity or fasting insulin; glucose tolerance ↓	/	[38]
Ace-K	Pure	40 or 120 mg/kg bw/day	Animal	Normal rats	28 days	No significant change in α-diversity; limited β-diversity/taxonomic changes	Conjugated bile acids altered; fecal metabolome minimally altered	No overt disease phenotype	/	[66]
Ace-K	Pure	37.5 mg/kg bw/day	Animal	Normal male/female mice	4 weeks	Male: *Bacteroides* ↑, *Anaerostipes* ↑, *Sutterella* ↑; *female*: *Lactobacillus* ↓, *Clostridium* ↓, *Mucispirillum* ↑	Sex-specific carbohydrate/LPS pathways and fecal metabolites altered	Body weight gain ↑ in males; no significant change in females	/	[22]
NHDC	Pure	5 mg/kg bw/day	Animal	Normal mice	11 weeks	No significant change in α-diversity or overall microbial composition	No significant change in AhR ligands	No significant change in host metabolic outcomes	/	[36]
NHDC	Pure	40 or 80 mg/kg bw/day	Animal	HFD rats	12 weeks	β-diversity altered; Proteobacteria ↓; Firmicutes/Bacteroidetes ratio ↓; *Bifidobacterium* ↑; *Clostridium* ↑; *Oscillospira* ↑; *Prevotella* ↓	Acetate ↑; propionate ↑; butyrate ↑	Fasting glucose ↓; fasting insulin ↓; serum triglycerides ↓; hepatic steatosis ↓; intestinal barrier function ↑	/	[70]
Neotame	Pure	2 mg/mL or 0.006 mg/mL	ex vivo	Healthy human fecal microbiota	24 h	Limited donor-specific taxonomic responses	Limited global metaproteomic change	/	/	[60]
Neotame	Pure	0.75 mg/kg bw/day	Animal	Normal mice	4 weeks	α-diversity ↓; β-diversity altered; Bacteroidetes ↑; *Blautia* ↓; *Dorea* ↓; *Oscillospira* ↓; *Ruminococcus* ↓	Butyrate synthesis genes ↓; fecal fatty acid/cholesterol metabolites altered	No significant change in body weight, food intake or behavior	/	[73]
Neotame	Formulated food	3 biscuits/day	Human crossover RCT	Adults with overweight/obesity	Acute + 2 weeks	/	/	Postprandial insulin iAUC ↓; no significant change in appetite hormones; gastrointestinal symptoms ↑	/	[74]
Cyclamate	Pure	1.6 mg/mL	Ex vivo	Healthy human fecal microbiota	24 h	Limited donor-specific taxonomic responses	Limited global metaproteomic change	/	/	[60]
Cyclamate	Pure	50–500 mg/kg bw/day	Animal + in silico	Normal rats	8 weeks	/	/	Cardiac inflammation/oxidative stress/apoptosis ↑; cardiac injury markers ↑	/	[75]
Mixed NAS	Multiple NAS; separate exposures	aspartame 1.36 mM; sucralose 25.2 mM; saccharin 2.72 mM	Single-strain/reporter assay	*Escherichia coli*, *Pseudomonas aeruginosa and Chromobacterium violaceum*	16–21 h	/	AHL quorum sensing ↓; LasR activity ↓; swarming motility ↓; violacein production ↓	/	/	[54]
Mixed NAS	Sucralose + Ace-K	sucralose 1.5 mg/mL + Ace-K 2.5 mg/mL	Animal	Normal mice	2 weeks	Microbial richness ↓; no significant change in β-diversity or individual taxa	/	Fasting glucose ↑; jejunal glucose absorption ↑; GLP-1 ↑	Antibiotic depletion showed microbiota-independent glucose absorption and partial microbiota-dependent GLP-1 response	[76]
Mixed NAS	Sucralose + Ace-K	ADI1× or ADI2× sucralose + Ace-K	Animal	Maternal-exposure mice/offspring	Gestation and lactation	Firmicutes ↑; *Akkermansia muciniphila* ↓	Fecal/plasma amino-acid metabolites altered	offspring body weight ↓; fasting glucose ↓; hepatic detoxification pathways ↓	/	[77]
Mixed NAS	Mixed S&SE products	ad libitum S&SE product substitution	Human RCT	Adults with overweight/obesity	10 months	No between-group change in α-diversity; β-diversity altered; *Prevotella* ↑; *Alloprevotella* ↑; *Butyricimonas* ↑; *Oscillospira* ↑	Methanogenesis ↑; SCFA fermentation pathways ↑	Weight regain ↓; no significant change in cardiometabolic markers	/	[13]
Mixed NAS	Mixed dietary exposure	50–100 mg/day	Human RCT	Adults with functional gastrointestinal symptoms	5 weeks	/	/	Diarrhea ↑; postprandial discomfort ↑; constipation ↑; no significant change in body weight/BMI	/	[78]

Abbreviations used: Ace-K, acesulfame potassium; ADI, acceptable daily intake; AHL, acyl-homoserine lactone; AhR, aryl hydrocarbon receptor; BMI, body mass index; bw, body weight; DCA, deoxycholic acid; DSS, dextran sulfate sodium; FMT, fecal microbiota transplantation; FOS, fructo-oligosaccharides; FXR, farnesoid X receptor; GLP-1, glucagon-like peptide-1; GPR43, G-protein-coupled receptor 43; HFD, high-fat diet; HFS, high-fat/high-sugar; IBD, inflammatory bowel disease; ICI, immune checkpoint inhibitor; iAUC, incremental area under the curve; IL-6, interleukin-6; iNOS, inducible nitric oxide synthase; KO, knockout; LasR, quorum-sensing transcriptional regulator LasR; LDL, low-density lipoprotein; LH, luteinizing hormone; LPS, lipopolysaccharide; MMP-2, matrix metalloproteinase-2; NAS, non-nutritive sweeteners; NHDC, neohesperidin dihydrochalcone; NNS, non-nutritive sweeteners; PD-1, programmed cell death protein 1; RCT, randomized controlled trial; SCFA, short-chain fatty acid; SIHUMIx, simplified human intestinal microbiota; S&SE, sugar- and sweetener-enhanced/sugar and sweetener-containing products; T1R2-KO, taste receptor type 1 member 2 knockout; T2D, type 2 diabetes; WT, wild-type. ↑ Increase; ↓ decrease.

## Data Availability

No new experimental, clinical, animal, or participant-level data were generated in this study. The bibliometric analysis and NAS-MAP framework were derived from published literature as described in Section 2. Bibliographic records obtained from Web of Science and Scopus remain subject to the respective database access conditions.

## Abbreviations

The following abbreviations are used in this manuscript:

Ace-K	Acesulfame potassium
ADI	Acceptable daily intake
AHL	Acyl-homoserine lactone
AhR	Aryl hydrocarbon receptor
AI	Artificial intelligence
ALP	Alkaline phosphatase
BMI	Body mass index
bw	Body weight
DCA	Deoxycholic acid
DSS	Dextran sulfate sodium
EFSA	European Food Safety Authority
F/B ratio	Firmicutes/Bacteroidetes ratio
FDA	U.S. Food and Drug Administration
FMT	Fecal microbiota transplantation
FOS	Fructo-oligosaccharides
FXR	Farnesoid X receptor
GALT	Gut-associated lymphoid tissue
GLP-1	Glucagon-like peptide-1
GLP-1R	Glucagon-like peptide-1 receptor
GLP-2R	Glucagon-like peptide-2 receptor
GPR43	G-protein-coupled receptor 43
GRAS	Generally Recognized as Safe
GRN	GRAS Notice
HFD	High-fat diet
HFS	High-fat/high-sugar
IBD	Inflammatory bowel disease
ICI	Immune checkpoint inhibitor
IFN-γ	Interferon-γ
iAUC	Incremental area under the curve
IL-1β	Interleukin-1β
IL-6	Interleukin-6
IL-10	Interleukin-10
iNOS	Inducible nitric oxide synthase
JECFA	Joint FAO/WHO Expert Committee on Food Additives
KO	Knockout
LasR	Quorum-sensing transcriptional regulator LasR
LDL	Low-density lipoprotein
LH	Luteinizing hormone
LPS	Lipopolysaccharide
MAdCAM-1	Mucosal addressin cell adhesion molecule-1
MMP-2	Matrix metalloproteinase-2
MW	Molecular weight
NAFLD	Nonalcoholic fatty liver disease
NAS	Non-nutritive artificial sweeteners / non-nutritive sweeteners
NAS-MAP	Non-nutritive artificial sweetener–microbiota-associated phenotype knowledge graph
NCD-RisC	NCD Risk Factor Collaboration
NHDC	Neohesperidin dihydrochalcone
NNS	Non-nutritive sweeteners
PD-1	Programmed cell death protein 1
QA	Question-answering
RCT	Randomized controlled trial
Reb D	Rebaudioside D
Reb M	Rebaudioside M
ROS	Reactive oxygen species
SCFA	Short-chain fatty acid
SHIME	Simulator of the Human Intestinal Microbial Ecosystem
SIFR	Systemic Intestinal Fermentation Research platform
SIHUMIx	Simplified human intestinal microbiota
S&SE	Sugar and sweetener-enhanced / sugar and sweetener-containing products
T1R2-KO	Taste receptor type 1 member 2 knockout
T2D	Type 2 diabetes
TNF-α	Tumor necrosis factor-α
UGT91D2	UDP-glycosyltransferase 91D2
WHO	World Health Organization
WT	Wild-type
